# Special Issue: Present and Future of Personalised Medicine for Endocrine Cancers

**DOI:** 10.3390/jpm12050710

**Published:** 2022-04-29

**Authors:** Cristina L. Ronchi, Barbara Altieri

**Affiliations:** 1 Institute of Metabolism and System Research, University of Birmingham, Birmingham B15 2TT, UK; 2 Centre for Endocrinology, Diabetes, and Metabolism (CEDAM), Birmingham Health Partners, Birmingham B15 2TT, UK; 3 Division of Endocrinology and Diabetes, Department of Internal Medicine I, University Hospital, University of Würzburg, 97080 Wuerzburg, Germany; altieri_b@ukw.de

Major technological advances in genomics have made it possible to identify critical genetic alterations in cancer, rendering oncology well along the path to personalised cancer medicine [1]. Thanks to developments in high-throughput genetics, several mutations and gene rearrangements have been identified in patients with endocrine cancers (e.g., thyroid and adrenocortical carcinoma [2,3,4]). This is particularly important when talking about targeted anticancer drugs that, contrary to standard chemotherapy, aim at one or more definite molecular pathway on cancer cells—so their selection is underlying patient’s genetic information. In fact, new affordable individual genomic analyses, as well as the opportunity to test new compounds in primary cells, may allow a personalised management of patients with endocrine malignancies [5,6,7]. This approach may improve the prediction of clinical outcome and therapeutic effectiveness, as well as help to avoid the use of ineffective drugs. However, further efforts are needed to obtain an adjustment of clinical management in patients with endocrine cancers that would rely solely or in great part on molecular pattern.

The aim of this Special Issue entitled “Present and Future of Personalised Medicine for Endocrine Cancers” was to offer an overview of exciting new research in the area of endocrine tumours that may set the stage for an innovative personalised management and future precision medicine modalities for individualised care. This issue encompasses nine publications on basic, translational and clinical research in different types of endocrine malignancies, including thyroid cancer [8,9,10,11], adrenocortical neoplasms [12,13,14], pheochromocytoma/paraganglioma [15] and pituitary tumours [16].

Looking across diseases, some themes are recurrent, such as the efforts to identify effective biomarkers useful to improve differential diagnosis and/or prognostication of endocrine cancers [11,12,14,15] or to predict response to treatment [10,15]. More specifically, in the field of thyroid cancers, Piciu et al. evaluated the correlation among different prognostic factors in papillary thyroid cancer, including the mutation of the BRAF V600E oncogene and the pathological standardized uptake values at the F18-fluorodeoxyglucose (FDG) positron emission tomography/computed tomography (PET/CT) [9]. In addition, Ieni and colleagues reviewed the role of molecular variability and intra-tumoral heterogeneity for the prognostic classification and clinical management of patients with differentiated thyroid cancer (DTC) [11]. Finally, another review by Feola et al. provided an excellent overview of the predictive clinical, biochemical and molecular factors for the response to treatment with the multikinase inhibitors sorafenib and lenvatinib in radioactive iodine refractory DTC. In this setting, most promising biomarkers are those involved in the angiogenic pathways [10].

Considering adrenocortical tumours, the role of several immunohistochemical markers for the distinction of carcinomas (ACC) from adenomas (ACA) and for prognostic stratification of malignant tumours have been investigated by Angelousi and colleagues, demonstrating that altered reticulin pattern and p53/Ki-67 expression are useful markers for the differential diagnosis of adrenocortical tumours [14]. Another study showed that cell-to-matrix-related molecules are specifically altered in ACC when compared to ACAs and identified osteopontin and HAS-1 (Hyaluronan Synthase 1) as novel potential diagnostic and prognostic biomarkers, respectively [12].

Regarding phaeochromocytoma and paraganglioma, which rare neuroendocrine tumours with uncertain malignant potential, Winzeler et al. extensively reviewed the relevant impact of molecular profiling for the understanding of the pathogenic mechanisms and for the prognostic classification, providing current and future opportunities for precision oncology [15].

From a different point of view, Koot and team underlined the importance of considering patients’ needs, preferences and values in enabling us to improve doctor–patient communication and to develop decision support tools in DTC [8]. Basile et al. performed a multicentre retrospective analysis on ACC patients treated with adjuvant mitotane, showing that extending the duration of the treatment over two years is not beneficial for patients with low to moderate risk of recurrence [13]. Finally, Duhamel and colleagues reported two case reports and a review of the literature on the efficacy of immunotherapy in aggressive pituitary tumours [16].

In general, there is agreement that further studies aimed at evaluating diagnostic, prognostic and predictive markers for therapeutic response are needed for tailoring patient management and allowing more appropriate treatment choices. We hope that future investigations, integrating modern molecular methodologies across cell and tissue models, computational approaches and prospective clinical trials, will continue to drive progress towards improved personalised management of endocrine cancers.
